# Influence of menstrual pain and symptoms on activities of daily living and work absenteeism: a cross-sectional study

**DOI:** 10.1186/s12978-024-01757-6

**Published:** 2024-02-19

**Authors:** Fatima Leon-Larios, Isabel Silva-Reus, María José Puente Martínez, Abel Renuncio Roba, Eva Ibeas Martínez, Isabel Lahoz Pascual, Maria Cassia Naranjo Ratia, José Cruz Quílez Conde

**Affiliations:** 1 Nursing Department, University of Seville, 41004 Seville, Spain; 2 Unidad de Salud Sexual y Reproductiva de Villena, 03400 Alicante, Spain; 3 Hospital San Pedro, 26006 Logroño, Spain; 4 Unidad de Atención a La Mujer Servicio de Obstetricia y Ginecología, Hospital Universitario de Burgos, 09006 Burgos, Spain; 5 Unidad de Salud Sexual y Reprodutiva de Alfaz del Pi, 03580 Alicante, Spain; 6 Hospital Clínico Universitario Zaragoza, 50009 Saragossa, Spain; 7 Centro de Salud Doctor Tolosa Latour. Chipiona, 11550 Cádiz, Spain; 8 Hospital Universitario de Basurto, 48013 Bilbo, Spain

**Keywords:** Menstrual leave, Painful menses, Dysmenorrhea, Work leave, Menstruation

## Abstract

**Objective:**

To examine the prevalence of menstrual pain among women of reproductive age and its impact on their daily lives and professional responsibilities.

**Methods:**

A cross-sectional and descriptive study was conducted in July and August 2022. Phone interviews were carried out using a random system to select women aged between 15 and 49 years old. The questionnaire included sociodemographic variables, contraception method used, characteristics of the menstrual pattern (pain and bleeding amount), its influence on their working life, and if they would need to resort to sick leaves due to the impairments arising from the menstrual symptoms.

**Results:**

A total of 1800 women representative of the Spanish population took part in this study. 72.6% of them report menstrual pain, with 45.9% requiring medication. 35.9% identify their menstrual bleeding as intense or very intense. 38.8% assert that menstrual discomforts affect their everyday life. 34.3% would have required not attending their work activities or having requested sick leave due to the discomforts, although only 17.3% of the women finally requested so, mainly because 58.4% considered that it might imply consequences in their professional environment, especially those with Higher Education. The women who report more discomfort are the youngest ones and those who resort to condoms as a contraceptive method (p < 0.001).

**Conclusions:**

Menstrual pain is a prevalent problem among women of reproductive age and can affect their everyday life and professional environment, requiring work leaves on some occasions.

## Plain language summary

This study is a starting point to know the prevalence of menstrual discomforts among Spanish women of reproductive age and their effect on the professional environment, to enable an assessment of the possible impact of the menstrual leave legislation recently implemented in Spain.

## Background

Since June 1st, 2023, Spain has become the first country in the European Union to grant the right to state-paid medical leave for menstrual pain from the onset of symptoms. Before this law was enacted, any woman experiencing menstrual pain severe enough to hinder her from performing her professional duties could take temporary, unpaid sick leave up until the third day [1]. With this new policy, menstruating women who experience symptoms related to their menstrual cycle that prevent them from properly carrying out their work tasks are entitled to take time off with pay.

Menstrual pain (dysmenorrhea) is extremely common in young women: between 30 and 90% have had painful menses on some occasions, with very severe pain in 10% to 20% of the cases [2, 3]. Dysmenorrhea is a prevalent problem that affects a percentage of women of reproductive age with a remarkable impact on their life and health [4]. Younger and nulliparous women are among those that can most undergo this situation [5, 6]. In addition, certain gynaecological problems such as endometriosis carry with them intense dysmenorrhea, exerting a negative impact on work attendance and ability [7].

Previous studies have indicated that pain can be so disabling as to even affect the performance of the activities of daily living [8]. Along with menstrual pain, it is usual for women to experience other menstrual symptoms that can affect their productive performance, with the consequent need to assess the impact of menstruation on work [9]. The data indicated that more than one-fourth of the women reduced their working hours or failed to attend work at least 1 day in the last 6 months due to painful menses [9]. This points to the fact that dysmenorrhea may not only be considered a health problem but also a social one, as it exerts an influence on health-related quality of life [10].

This Law is already a reality in other Asian countries such as Japan, China, South Korea, Taiwan, and Zambia, where the prevalence of dysmenorrhea among women and how it affects their performance at work have already been studied; however, there are no studies about the use of menstrual leave, the population's attitude towards the Law or the socio-political implications [11]. In addition, various companies and professional organizations from the United Kingdom, Australia, and Iran have also chosen to implement menstrual leave, although there are no available data about the actual impact of this measure on female workers [12, 13]

The arguments in favour of this Law are related to the fact that this menstrual leave contributes to menstrual health by providing time to rest, recover, and/or seek help from a professional. However, counterarguments have also been identified, as this Law might be contributing to the stigmatization of menstruating women [11].

The purpose of this study was to examine the prevalence of menstrual pain among women of reproductive age and its impact on their daily lives and professional responsibilities.

## Methods

### Study design and participants

A cross-sectional and descriptive study was conducted in July and August 2022.

The study population was comprised of women aged from 15 to 49 years old. The average age of sexual initiation, often referred to as 'sexarche', is approximately 15 years old, which is why it has been established as the minimum age threshold [14]. According to the updated data available, this population group accounts for 11,267,550 women in Spain, which represents 57.5% of the total female population and 24.2% of the entire Spanish population. The study’s geographical scope is nationwide, thus allowing the representativeness of the sample. The questionnaire was applied to 1,800 women representative of the entire Spanish population. The distribution of the sample was proportional to the actual distribution of the population with an adjustment system by age quotas, with a random last selection of the interviewees. Specifically, the sample size proposed provided a statistical error for global data of ± 2.31% for a 95.5% confidence level (Two Sigma) and a population distribution of p/q = 50/50.

The interviews were conducted following a mixed methodology: via telephone calls, using the CATI (Computer Assisted Telephone Interviewing) system, and online, by using the CAWI (Computer Assisted Web Interviewing) system. Using this mixed methodology allowed for optimizing access to population segments that are increasingly more difficult to reach via telephone calls and are usually under-represented in samples. They are mostly young people who either have no landline, are very hard to locate on family landlines, or tend not to answer telephone calls on their mobile devices. This technique ensured maximum efficiency in research studies of a sociological nature by combining its ability to target large population groups, attain full geographical amplitude and diversification, and work with complex questionnaires. The entire survey process was in charge of the Sigma Dos company, which specializes in digital marketing.

### Instrument

A telephone-based structured questionnaire comprised of questions about sociodemographic variables: age group (15–19 years old; 20–24 years old; 25–20 years old; 30–34 years old; 25–39 years old; 40–44 years old; 45–49 years old), marital status (living with partner; with steady partner, not living together; no steady partner), children (yes/no), nationality (born in Spain; born abroad, Spanish nationality; born abroad, non-Spanish nationality), schooling level (Less than elementary school; elementary school; high school; pre-University higher education; university studies), family income per month (Less than € 1100; from € 1100 to € 1800; from € 1801 to € 2700; from € 2701 to € 3900; more than € 3900), work situation (freelance; public administration, private company (fixed job position); private company (eventual worker); unemployed and looking for a job; student; housewife; retired o disabled) and about sexual and contraceptive habits (contraceptive methods used), as well as related to menstruation (if use of hormonal contraception for pain, for bleeding, for both reasons, for other reasons), bleeding duration (in days) and amount (scarce, moderate, intense, very intense)) and pain (no; mild pain; moderate pain; intense pain)). In addition to its repercussions in their daily activities (none; it does not limit me; it limits me to lead a normal life; it prevents me from leading a normal life) and professional activities (requested sick leave (yes/no) and had consequences on work of this sick leave due to menstruation (yes/no)) [14].

### Statistical analysis

Descriptive statistics were used to determine the prevalence of painful menses among the participants. The participants’ characteristics were reported using frequencies and percentages. The relationship between sociodemographic variables menstrual signs and symptoms and work-related variables was reported using frequencies, percentages, and the x^2^ test. Cramer´s V was calculated for effect sizes.

A threshold of p < 0.05 was established to consider an association significant. The statistical analyses were performed in IBM SPSS Statistics for Windows, version 26 (IBM Corp., Armonk, NY, USA).

## Results

### Characteristics of the participants

A total of 1,800 participants were included in the study. Table 1 shows the sociodemographic characteristics of the sample corresponding to the average profile of women of reproductive age in Spain. Most of the women were born in Spain, belong to different age groups, have a partner (although not always living together) and slightly more than half of them have not had any children.

**Table 1:** Characteristics of the study participants

Variables	Categories	N	%
Age	15–19 years old	201	11.2
	20–24 years old	200	11.1
	25–29 years old	214	11.9
	30–34 years old	238	13.2
	35–39 years old	279	15.5
	40–44 years old	333	18.5
	45–49 years old	335	18.6
Origin	Born in Spain	1,501	84.4
	Born abroad; Spanish nationality	171	9.6
	Born abroad; non-Spanish nationality	106	6.0
Marital status	Living with partner	815	54.5
	With steady partner, not living together	324	21.7
	No steady partner	357	23.9
Children	Yes	789	44.8
	No	971	55.2
Family income per month	Less than € 1,100	277	17.7
	From € 1,100 to € 1,800	523	33.5
	From € 1,801 to € 2,700	433	27.7
	From € 2,701 to € 3,900	231	14.8
	More than € 3,900	98	6.3
Schooling level	Less than Elementary School	2	0.1
	Elementary School	87	4.9
	High School	557	31.3
	Pre-University Higher Education	428	24.0
	University Studies	706	39.7
Work situation	Freelance	162	6.2
	Public Administration	196	7.5
	Private Company (Fixed job position)	848	32.4
	Private Company (Eventual worker)	848	32.4
	Unemployed and looking for a job	177	6.8
	Student	277	10.6
	Housewife	90	3.4
	Retired or disabled	22	0.8

As can be seen in Table 2, when we asked about the usual signs and symptoms associated with the participants' menstruation, 72.6% asserted that their menses were painful and defined bleeding as intense or very intense in 35.9% of the cases. The pain reported by the women can even affect their daily life and performance of their duties in 38.8% of the cases, with a mean of 2.95 days affected by this condition and requiring pain relief medications in 45.9%. At least 34.3% had to request sick leave or not attend work at some moment due to menstrual pain or bleeding; however, only 17.3% confirm that they have eventually requested leaves due to this reason. More than half of the women think that requesting sick leave for this reason could lead to negative work outcomes, such as being fired.

**Table 2:** Menstrual profile of the participants

Variables	Categories	N	%
Menstrual pain	No	482	27.4
	Mild pain (Not requiring medication)	471	26.7
	Moderate pain (Requiring medication)	429	24.3
	Intense pain (Requiring medication)	381	21.6
Menstruation bleeding amount	Scarce	259	15.0
	Moderate	847	49.1
	Intense	434	25.2
	Very intense	184	10.7
Repercussion of menstrual pain on activities of daily living	None, it does not limit me	1065	61.2
	It limits me to lead a normal life	549	31.5
	It prevents me from leading a normal life	127	7.3
Have you ever required sick leave or not attending your activity due to pain and/or bleeding?	Yes	602	34.3
	No	1151	65.7
Have you ever requested sick leave or not attended your activity due to pain and/or bleeding?	Yes	303	17.3
	No	1450	82.7
Do you believe that requesting sick leave due to menstruation can have work-related consequences?	Yes	966	58.4
	No	689	41.6

	N	Mean	SD
Days a month that pain or bleeding limits you or prevents you from leading a normal life	676	2.95	1.97

Table 3 shows the characteristics associated with the participants' menstruation according to age groups. We can see that the youngest women are the ones reporting more menstrual pain and bleeding amount. In addition, they refer to more limitations to performing their usual activities during the menstrual period, even with the need to request sick leave or not to attend work due to pain and/or bleeding. It is noticed that this entire symptomatology and limitations are reduced as the age group increases (p < 0.01). Nevertheless, no statistically significant differences are observed between the women belonging to the different age groups and the repercussions that requesting sick leave due to menstruation-related reasons might have on their professional lives.

**Table 3:** Menstrual profile according to age groups

Variables	Categories	15–19 years old	20–24 years old	25–29 years old	30–34 years old	35–39 years old	40–44 years old	45–49 years old	p-value
Menstrual pain	No	14.6^b^	13.8^b^	20.8^a^	21.1^a^	27.3	32.7^a^	46.7^b^	Chi^2^ = 137.38; DoF = 18; p = 0.0; V-Cramer = 0.161
	Mild pain	26.6	25.0	26.9	34.6^b^	30.6	26.6	18.7^b^	
	Moderate pain	33.9^b^	38.3^b^	28.3	21.5	21.6	22.0	14.3^b^	
	Intense pain	25.0	23.0	24.1	22.8	20.5	18.7	20.2	
Menstruation bleeding amount	Scarce	5.7^b^	10.8	9.2^a^	18.7	12.1	21.4^b^	20.6^b^	Chi^2^ = 55.09; DoF = 18; p = 0.00001; V-Cramer = 0.103
	Moderate	53.1	50.0	56.0	46.8	51.3	46.3	44.2	
	Intense	32.3^a^	28.9	27.1	22.1	24.9	20.2^a^	24.9	
	Very intense	8.9	10.3	7.7	12.3	11.7	12.1	10.3	
Repercussion of menstrual pain on activities of daily living	None, it does not limit me	55.0	45.6^b^	54.3^a^	60.0	61.7	67.9^a^	72.4^b^	Chi^2^ = 55.13; DoF = 12; p = 0.0; V-Cramer = 0.126
	It limits leading a normal life	36.0	40.9^b^	38.5^a^	32.8	32.5	25.7^a^	22.9^b^	
	It precludes leading a normal life	9.0	13.5^b^	7.2	7.2	5.8	6.4	4.8	
Have you ever required sick leave or not attending your activity due to pain and/or bleeding?	Yes	50.5^b^	51.5^b^	44.2^b^	33.2	30.7	25.2^b^	21.7^b^	Chi^2^ = 93.68; DoF = 6; p = 0.0; V-Cramer = 0.361
	No	49.5^b^	48.5^b^	55.8^b^	66.8	69.3	74.8^b^	78.3^b^	
Have you ever requested sick leave or not attended your activity due to pain and/or bleeding?	Yes	36.4^b^	31.9^b^	17.7	12.5^a^	13.9	11.0^b^	10.2^b^	Chi^2^ = 103.00; DoF = 6; p = 0.0; V-Cramer = 0.242
	No	63.6^b^	68.1^b^	82.3	87.5^a^	86.1	89.0^b^	89.8^b^	
Do you believe that requesting sick leave due to menstruation can have work-related consequences?	Yes	53.7	64.3	55.8	61.7	63.5	57.6	53.4	Chi^2^ = 11.85; DoF = 6; p = 0.066; V-Cramer = 0.085
	No	46.3	35.7	44.2	38.3	36.5	42.4	46.6	

In Table 4**,** the results indicate that experiencing painful menstruation has a significant effect on daily activities, χ^2^(3) = 454, p < 0.001, R2 Nagelkerke = 0.193, with a stronger effect when the pain is severe and requires medication (Z = 17.42, p < 0.001). Similarly, having painful menstruation is associated with the need to request sick leave, χ^2^(3) = 251, p < 0.001, R2 Nagelkerke = 0.186, especially with severe pain (Z = 13.69, p < 0.001). Finally, an impact on the probability of requesting sick leave is also observed, χ^2^(3) = 129, p < 0.001, R2 Nagelkerke = 0.119, with a higher incidence when there is more pain (Z = 9.52, p < 0.001).

**Table 4:** Relationship among menstrual pain, daily lives, and professional responsibilities

	Χ^2^	R^2^_N_	Predictor	Estimate	SE	Z	OR
DV: Repercussion of menstrual pain on activities of daily living	454***	0.193					
			Scarce pain—No	1.13	0.18	6.20***	3.08
			Moderate pain– No	2.02	0.18	11.39***	7.50
			Intense pain—No	3.24	0.19	17.42***	25.52
DV: Have you ever required sick leave or not attending your activity due to pain and/or bleeding?	251***	0.186					
			Scarce pain—No	0.91	0.17	5.28***	2.48
			Moderate pain– No	1.61	0.17	9.59***	5.02
			Intense pain—No	2.36	0.17	13.69***	10.61
DV: Have you ever requested sick leave or not attended your activity due to pain and/or bleeding?	120***	0.119					
			Scarce pain—No	0.66	0.24	2.79**	1.93
			Moderate pain– No	1.28	0.22	5.78***	3.60
			Intense pain—No	2.05	0.22	9.52***	7.75

IV Menstrual pain

^***^ p < .001

^**^ p < .01

Table 5 shows that there are no differences in the women's menstrual profile according to their schooling level but, rather, in the repercussions they consider that having to request sick leaves due to the symptoms associated with the menstrual period might have on their professional life. The women with university studies have requested sick leaves due to this reason to a lesser extent and consider that taking them would have work-related repercussions.

**Table 5:** Influence of the menstrual profile on work according to schooling level

Variables	Categories	Less than Elementary School	Elementary School	High School	Other non-university studies	University Studies	Sig
Menstrual pain	No	0.0	25.3	25.2	25.8	29.8	Chi^2^ = 9.43; DoF = 12; p = 0.666; V-Cramer = 0.042
	Mild pain	50.0	25.3	26.3	28.8	26.0	
	Moderate pain	0.0	28.9	24.8	23.2	24.4	
	Intense pain	50.0	20.5	23.7	22.2	19.8	
Menstruation bleeding amount	Scarce	0.0	23.2	14.4	13.9	15.2	Chi^2^ = 19.00; DoF = 12; p = 0.088; V-Cramer = 0.061
	Moderate	100.0	42.7	46.4	49.8	51.5	
	Intense	0.0	15.9	28.0	26.9	23.2	
	Very intense	0.0	18.3	11.2	9.4	10.2	
Repercussion of menstrual pain on activities of daily living	None, it does not limit me	50.0	71.6	59.7	58.7	62.5	Chi^2^ = 8.82; DoF = 8; p = 0.358; V-Cramer = 0.050
	It limits leading a normal life	50.0	19.8	33.7	33.7	30.0	
	It precludes leading a normal life	0.0	8.6	6.6	7.6	7.5	
Have you ever required sick leave or not attending your activity due to pain and/or bleeding?	Yes	50.0	16.7^b^	39.8^b^	34.1	32.6	Chi^2^ = 19.92; DoF = 4; p = 0.00052; V-Cramer = 0.107
	No	50.0	83.3^b^	60.2^b^	65.9	67.4	
Have you ever requested sick leave or not attended your activity due to pain and/or bleeding?	Yes	100.0^a^	9.5	22.9^b^	17.9	13.4^b^	Chi^2^ = 28.07; DoF = 4; p = 0.00001; V-Cramer = 0.127
	No	0.0^a^	90.5	77.1^b^	82.1	86.6^b^	
Do you believe that requesting sick leave due to menstruation can have work-related consequences?	Yes	50.0	57.7	57.6	59.4	58.7	Chi^2^ = 0.43; DoF = 4; p = 0.980; V-Cramer = 0.006
	No	50.0	42.3	42.4	40.6	41.3	

Cell significance: a: p < 0.05; b: p < 0.05

Table 6 shows that the women with more intense menstrual pain and/or bleeding resort more to some hormonal contraceptive method as treatment. Half of the women who requested sick leave or not attending their activities due to pain were already on hormonal contraception due to menstrual pain and abundant bleeding. The women who asserted more limitations to developing their daily activities are those who take more hormonal contraceptives, especially for pain, as well as those who have already requested sick leave due to this reason.

**Table 6:** Use of contraceptive methods as treatment and menstrual signs/symptoms

			**Do you use hormonal contraception?**				
		**No**	**Yes, for pain**	**Yes, for bleeding**	**Yes, for both reasons**	**Yes, for other reasons**	Sig.
Menstrual pain	No	28.2^b^	14.8^a^	15.3^a^	9.1^b^	37.7^a^	Chi^2^ = 43.19; DoF = 12; p = 0.0; V-Cramer = 0.106
	Mild pain	28.6	25.9	28.2	32.5	22.1	
	Moderate pain	24.7	28.7	34.1	24.7	19.5	
	Intense pain	18.5^b^	30.6^a^	22.4	33.8^b^	20.8	
Menstruation bleeding amount	Scarce	15.3	13.1	10.6	18.9	30.3^b^	Chi^2^ = 33.47; DoF = 12; p = 0.001; V-Cramer = 0.094
	Moderate	52.6^b^	49.5	37.6^a^	41.9	44.7	
	Intense	23.4	25.2	31.8	27.0	15.8	
	Very intense	8.7^a^	12.1	20.0^b^	12.2	9.2	
Repercussion of menstrual pain on activities of daily living	None, it does not limit me	64.8^b^	42.6^b^	51.8	43.4^b^	59.7	Chi^2^ = 43.39; DoF = 8; p = 0.0; V-Cramer = 0.131
	It limits leading a normal life	30.1^b^	41.7^a^	38.8	46.1^b^	29.9	
	It precludes leading a normal life	5.1^b^	15.7^b^	9.4	10.5	10.4	
Have you ever required sick leave or not attending your activity due to pain and/or bleeding?	Yes	33.9^b^	51.0^b^	34.9	46.7^a^	32.5	Chi^2^ = 16.10; DoF = 4; p = 0.003; V-Cramer = 0.113
	No	66.1^b^	49.0^b^	65.1	53.3^a^	67.5	
Have you ever requested sick leave or not attended your activity due to pain and/or bleeding?	Yes	16.4^b^	23.5	25.0	30.1^b^	16.9	Chi^2^ = 13.49; DoF = 4; p = 0.009; V-Cramer = 0.103
	No	83.6^b^	76.5	75.0	69.9^b^	83.1	
Do you believe that requesting sick leave due to menstruation can have work-related consequences?	Yes	57.3	65.0	53.9	66.2	59.4	Chi^2^ = 4.69; DoF = 4; p = 0.320; V-Cramer = 0.063
	No	42.7	35.0	46.1	33.8	40.6	

Cell significance: a: p < 0.05; b: p < 0.05

As can be seen in Table 7, the women who resort to condoms mostly stated having felt the need to request sick leave or not attending their work activity as a consequence of menstrual pain and/or bleeding.

**Table 7:** Type of contraceptive methods and work absenteeism

Contraceptive method	Have you ever required sick leave?	
	Yes (%)	p-value
Condoms	40.2	**p < 0.01**
Pills	35.3	
Intrauterine Device	22.3	
Vaginal ring	28.6	
Voluntary sterilization	22.2	
Other methods	38.7	
None	29	

## Discussion

The purpose of this study was to analyse the characteristics of the menstrual pattern in women of reproductive age in Spain, as well as the association between menstrual symptoms and their influence on work absenteeism or sick leave requests.

As the inaugural comprehensive national study of its kind in Spain, this research enables the assessment of the prevalence and factors associated with painful menstruation among Spanish women. A key focus of the study was to determine the proportion of women who view their menstrual pain as incapacitating enough to necessitate taking sick leave, particularly in light of Spain's recent introduction of Menstrual Leave Law. Additionally, the study aimed to investigate the determinants influencing women’s decisions to opt for or against taking menstrual sick leave.

In our study, 7 out of every 10 women had menstrual pain, which is consistent with other studies conducted with women of reproductive age where 4 and 6 out of every 10 women mentioned painful menses, respectively. The data from our research are in line with those found in similar surveys conducted in other countries, where 10% to 20% of the women were willing to request sick leave due to the associated symptoms [3, 15, 16]. However, more than half of these women are also aware that requesting this leave might have negative repercussions for them in their workplace [13]; this accounts for the actual percentage of women who finally requested temporary sick leaves due to menstrual symptoms being so low.

The women who mentioned usual menstrual pain were more willing to request sick leave due to this reason, as they felt that it had precluded them from leading a normal life at least once. This is in line with what was found in other studies, where the women who had more somatic symptoms related to menstruation were more willing to interrupt their work for this reason [5, 16, 17].

Younger women reported experiencing a higher frequency of menstruation-related symptoms, with a noted decrease in symptoms as age increases. Regarding the impact of menstrual issues on their professional lives, no significant concern was found to vary with age. These findings are consistent with the results presented in other research [3, 5]

This research aimed to investigate the adverse effects of menstrual symptoms on the professional lives of women. We examined how the educational levels of participants influenced their experience of menstrual symptoms and any corresponding work-related repercussions. Notably, women with higher education often perceived sick leave for menstrual discomfort as an obstacle to career progression. This concern is particularly acute due to the social stigma commonly associated with menstruation, a topic that merits further exploration according to other studies [11]. The pronounced apprehension among university-educated women, who are likely to occupy positions with greater responsibility, may be attributed to the fear of stigmatization and the negative implications for career advancement. These findings are consistent with previous research that recognized this as an influential factor, with women from lower socioeconomic backgrounds reporting more frequent work absences due to menstrual issues [15, 18].

In this research, menstrual pain has been related to the use of contraceptive methods, noticing that the highest painful menses rates are found among those women who resort to condoms as a contraceptive method. In other studies, it has been already shown that using contraceptive methods can reduce menstrual pain and improve women’s quality of life [9, 10, 19], although we should also acknowledge that dysmenorrhea can be the result of several factors such as nutrition, physical exercise, stress, sleep [20, 21] and even gynaecological problems such as endometriosis [7, 11, 22]. Therefore, it would be interesting to analyse it considering all these factors that have been described. In our study, the women who resorted to condoms as contraceptive methods had to resort more to sick leaves than those using hormonal methods. This is also in line with the result which indicates that the youngest women are the ones with the most menstrual symptoms, both pain and abundant bleeding; in addition, it is the contraceptive method most frequently used in this age group as well [14, 20]. In turn, this leads them to having to resort to work absences and/or to request sick leaves more frequently.

Spain's pioneering role in the European context for legislating menstrual leave has sparked a significant social discourse, with various perspectives both endorsing and opposing this policy, echoing debates from nations with established menstrual leave practices [12, 23, 24]. While recognizing menstrual leave represents progress for those who suffer from debilitating menstrual pain affecting their work capacity, it simultaneously brings to the fore concerns about reinforcing gender biases, potentially casting women as less productive in the workplace [25, 26]. Consequently, it is crucial to conduct more comprehensive research on the impact of this policy on women's health and to evaluate its broader implications and advantages [13].

Recognizing menstrual leave has the potential to diminish the stigma surrounding menstruation in professional settings. However, there is a risk that such policies might also contribute to workplace discrimination and the reinforcement of sexist norms and attitudes [23, 27], reflecting the viewpoint that menstruation is a private matter not to be disclosed at work [13, 28]. In an effort to mitigate negative workplace perceptions, some companies have implemented policies that enable women to telecommute during days when they are physically unwell. Additionally, the creation of designated 'well-being rooms' within the office environment has been adopted as a strategy, providing women with a tranquil space to focus on their tasks and personal comfort [11, 13].

Among the limitations of this research, we identified that, as it is a cross-sectional and descriptive study, we cannot establish causalities. On the other hand, this study only included cisgender women but it would be interesting to investigate if this measure would need to be extended to menstruators^1^ that do not identify themselves as women so that it became an inclusive measure. Being a study with a representative sample of the Spanish female population and selected using random sampling, it allows having an overview of the phenomenon to continue investigating in this line.

An interesting study prospect would be to assess the actual impact of this measure recently launched and to analyse the factors associated with women resorting to it or not, as well as the repercussions in their workplaces.

## Conclusions

Our study demonstrates that menstrual pain is a prevalent issue among women of reproductive age, with a higher incidence reported among younger women. This condition significantly impacts their daily and professional lives. While many women may have legitimate reasons to take time off from work due to menstrual discomfort, only a fraction avail of sick leave, possibly due to concerns over professional repercussions, a trend that is particularly evident among those with higher education. Furthermore, our findings suggest that those who experience greater menstrual discomfort are more likely to use male condoms as a method of contraception, as well as those in the younger age bracket.

## Data Availability

Data are available on reasonable request.

## Abbreviations

CATI: Computer-assisted telephone interviewing
CAWI: Computer assisted web interviewing

## Footnotes

1 menstruator: The term is used on purpose; it acknowledges that people who menstruate are not all women and that not all women menstruate.
